# Predictions of Daily Milk and Fat Yields, Major Groups of Fatty Acids, and C18:1 *cis*-9 from Single Milking Data without a Milking Interval

**DOI:** 10.3390/ani5030377

**Published:** 2015-07-31

**Authors:** Valérie M. R. Arnould, Romain Reding, Jeanne Bormann, Nicolas Gengler, Hélène Soyeurt

**Affiliations:** 1 CONVIS s.c., Zone Artisanale et Commerciale, 4, Ettelbruck, L-9085, Luxembourg; E-Mails: valerie.arnould@convis.lu (V.M.R.A.); romain.reding@convis.lu (R.R.); 2 Gembloux Agro Bio-Tech, Agriculture, Bio-engineering and Chemistry Department Animal Science and Nutrition Unit, University of Liège, Passage des Déportés 2, Gembloux B-5030, Belgium; E-Mail: nicolas.gengler@ulg.ac.be; 3 Administration des Services Techniques de l'Agriculture ASTA, Luxembourg L-1019, Luxembourg; E-Mail: jeanne.bormann@asta.etat.lu; 4 Gembloux Agro Bio-Tech, Department of Agricultural Science, Statistics, Informatics, and Applied Modeling Unit, University of Liège, Passage des Déportés 2, Gembloux B-5030, Belgium

**Keywords:** milk recording, fatty acid groups, prediction model, single milking

## Abstract

**Simple Summary:**

Reducing the frequency of milk recording decreases the costs of official milk recording. However, this approach can negatively affect the accuracy of predicting daily yields. Equations to predict daily yield from morning or evening data were developed in this study for fatty milk components from traits recorded easily by milk recording organizations. The correlation values ranged from 96.4% to 97.6% (96.9% to 98.3%) when the daily yields were estimated from the morning (evening) milkings. The simplicity of the proposed models which do not include the milking interval should facilitate their use by breeding and milk recording organizations.

**Abstract:**

Reducing the frequency of milk recording would help reduce the costs of official milk recording. However, this approach could also negatively affect the accuracy of predicting daily yields. This problem has been investigated in numerous studies. In addition, published equations take into account milking intervals (MI), and these are often not available and/or are unreliable in practice. The first objective of this study was to propose models in which the MI was replaced by a combination of data easily recorded by dairy farmers. The second objective was to further investigate the fatty acids (FA) present in milk. Equations to predict daily yield from AM or PM data were based on a calibration database containing 79,971 records related to 51 traits [milk yield (expected AM, expected PM, and expected daily); fat content (expected AM, expected PM, and expected daily); fat yield (expected AM, expected PM, and expected daily; g/day); levels of seven different FAs or FA groups (expected AM, expected PM, and expected daily; g/dL milk), and the corresponding FA yields for these seven FA types/groups (expected AM, expected PM, and expected daily; g/day)]. These equations were validated using two distinct external datasets. The results obtained from the proposed models were compared to previously published results for models which included a MI effect. The corresponding correlation values ranged from 96.4% to 97.6% when the daily yields were estimated from the AM milkings and ranged from 96.9% to 98.3% when the daily yields were estimated from the PM milkings. The simplicity of these proposed models should facilitate their use by breeding and milk recording organizations.

## 1. Introduction

According to Arnould *et al*. [1], milk yield, and, particularly, milk fat composition, may facilitate the development of strategies to prevent and monitor milk production dysfunction in dairy cattle, and may improve the sustainability of dairy production systems. Correspondingly, various milk fatty acids (FA) have shown a relationship with methane production in dairy cattle. For example, positive correlations between saturated FA (SFA) and methane output has been observed (r = 0.87–0.91) [2]. Another example involves ketosis detection. In reports by van Haelst *et al.* and Gross *et al.* [3,4]), a high proportion of long chain FA (LCFA; especially if combined with a lower proportion of medium chain FA (MCFA)), and especially a high proportion of C18:1 cis-9, in milk fat were found to be good predictors of subclinical ketosis. Therefore, a regular quantification of FA in milk is relevant.

Recent studies have demonstrated that mid-infrared spectrometry (MIR) has the potential to quantify the FA content of milk [5,6,7]). Therefore, the creation of spectral databases represents valuable resources for determining the FA profile of test-day samples collected from lactating cows that are routinely monitored using specific MIR calibration equations. For instance, this is currently realized by the Belgian (Walloon Breeding Association, Ciney, Belgium) and Luxembourg (CONVIS s.c., Ettelbruck, Luxembourg) milk recording organizations. Thanks to the easy acquisition of spectral data, other countries will realize the same work in a near future.

To develop robust management tools, the used phenotypic data should be homogenous. However, the uses of different sampling methods can bring heterogeneity. Milk recording organizations in many countries use more and more often an alternate morning (AM) and evening (PM) testing scheme since it is less expensive than analyzing one milk sample per cow that includes 50% of a representative AM milking fraction and 50% of a representative PM milking fraction. Since the 1970s, numerous equations have been evaluated for their capacity to estimate total daily yields for traditional production traits (*i.e*., milk, fat, and protein) from alternate protocols. For example, Lee and Wardrop [8] studied the effects of milking interval (MI; the duration between two consecutive milkings, expressed in h or min; AM or PM) and stage of lactation on daily milk, fat, and protein yields, and fat and protein content. In 1986, adjustment factors for daily milk, fat, and protein yields were reported by Delorenzo and Wiggans [9], and these remain the most widely used factors based on their ability to take into account heterogeneous means and variances between MIs and classes of days in milk (cDIM). In 2000, this model was modified [10], and the changes were approved by the International Committee for Animal Recording [11]. At our knowledge, nothing is done currently about FA. The general aim of this paper is therefore to develop equations to estimate the daily yields of the major FAs present in milk, including SFA, unsaturated FA (UFA), mono-unsaturated FA (MUFA), short-chain FA (SCFA), MCFA, and LCFA from a single milking. In addition, C18:1 *cis*-9 was also studied because this FA is interesting for management purposes [1].

Most of the studies mentioned in the above paragraph included an MI parameter in their predictive models. However, such information might be difficult to collect on a farm since the time and duration of milking is often inconsistent. In a previous report [12], it is mentioned that changes in milk composition can occur according to the MI primarily due to a dilution effect. Thus, a high volume of milk produced during one milking would be predicted to contain less fat and protein compared to a smaller volume of milk. Based on this concept, MIs could affect the levels of detected milk components. Therefore, an additional aim of the present study was to compare the results obtained using the models of Liu [10] and Berry [13] that include an MI effect for milk, fat, and protein yields with the results obtained from models that include only factors related to milk composition and production. Potentially, such models could provide a straightforward prediction of daily yields for production traits from more readily available information (*i.e*., fat and protein content and other MIR predicted traits).

## 2. Materials and Methods

### 2.1. Available Data

#### 2.1.1. Overall Strategy

To develop equations which permit the estimation of FA daily yields from one milking, measurements of milk yield and milk composition at each milking are needed, as well as milk composition data from 50% AM and 50% PM milk samples. Unfortunately, separate AM and PM milk samples at the same test day were never collected by the Luxembourg milk recording (CONVIS s.c., Ettelbruck, Luxembourg). Therefore, the innovative part of this study was to create a calibration set including AM and PM expected values estimated using selection index theory from available mixed samples. Then, the equations developed using these expected phenotypes were validated using real data. Indeed, a sampling including AM, PM and mixed milk samples was performed on a limited number of cows and herds in order to create a validation set. More details are given in the following sections.

#### 2.1.2. Calibration Data

The calibration dataset included milk samples collected in Luxembourg between October 2007 and April 2013 during routine conventional milk testing (data S). These milk samples were composed of 50% morning milk and 50% evening milk and were collected from 21,582 Holstein cows in 163 herds. All of the milk samples were analyzed by MIR spectrometry using a Foss MilkoScan FT6000 (Hillerod, Denmark) at CONVIS s.c. (Ettelbruck, Luxembourg). MIR analysis of the milk samples provided spectral data and the quantities of major milk components, including fat and protein content. By applying the updated equations of Soyeurt *et al.* [7], SFA, MUFA, UFA, SCFA, MCFA, LCFA, and C18:1 *cis-*9 content in each milk sample (g/L) were determined. As a result, the ratio of the standard error of cross-validation to the standard deviation (SD) of gas chromatography FA values used in the calibration set (referred to as a RPD parameter) greater than five was observed. Table 1 shows the statistical parameters of the calibration equations used. The data used to build the mid-infrared FA equations were not related to the data used in this study.

**Table 1:** Estimated statistical parameters for each calibration equation that estimated the concentration of fatty acids (FAs) in milk (g/dL of milk).

FA	*N*	*Mean*	*SD*	*SECV*	*R²cv*	*RPD*
SFA	1176	2.69	0.79	0.051	0.9958	15.34
MUFA	1180	1.04	0.34	0.047	0.9805	7.18
UFA	1179	1.20	0.39	0.051	0.9828	7.62
SCFA	1185	0.35	0.10	0.020	0.9613	5.10
MCFA	1187	2.06	0.65	0.086	0.9824	7.53
LCFA	1188	1.50	0.52	0.087	0.9718	5.96
C18:1 *cis*-9	1194	0.71	0.26	0.051	0.9610	5.06

FA = fatty acid; SD = standard deviation; SECV = standard error of cross-validation; R²cv = cross-validation coefficient of determination; RPD = ratio of standard error of cross-validation to standard deviation; SFA = saturated fatty acids; MUFA = monounsaturated fatty acids; UFA = unsaturated fatty acids; SCFA = short chain fatty acids, MCFA = medium chain fatty acids; LCFA = long chain fatty acids.

Records were discarded from the dataset if test-day records were lower or higher than mean ± three times the observed SD. Furthermore, only spectral data with known production factors such as DIM, and parity were kept. After these edits, the final calibration dataset contained 79,971 records.

Data from the S milk recording scheme included observed FAT_50/50_, SFA_50/50_, MUFA_50/50_, UFA_50/50_, SCFA_50/50_, MCFA_50/50_, LCFA_50/50_, and C18:1 *cis-9*_50/50_. This dataset also contained AM, PM, and 24 h milk yields. However, this dataset did not contain records for milk composition related to AM or PM milkings. Therefore, a method similar to that of the selection index theory was used to calculate expected values for: SFA_AM,_ SFA_PM_, MUFA_AM,_ MUFA_PM_, UFA_AM_, UFA_PM_, SCFA_AM_, SCFA_PM_, MCFA_AM_, MCFA_PM_, LCFA_AM_, LCFA_PM_, and C18:1 *cis-9*_AM_, C181 *cis-9*_PM_. This method was based on a linear combination of phenotypic data and the following two equations:

(1) $$observed\_{trait}_{\text{50/50}}=\text{0.5}\times {value}_{AM}+\text{0.5}\times {value}_{PM}$$

(2) $$\text{exp }ected\_{trait}_{\text{AM or PM}}=f(milk\_{yield}_{\text{AM or PM}},fat\_{yield}_{\text{AM or PM}})$$

Equation (1) assumes that the milk samples contained 50% AM milk and 50% PM milk. In addition, a non-zero correlation between milk fat composition during the AM or PM milking and the milk and fat yields during the same milking were also assumed (Equation (2)).

Equations (1) and (2) can then be combined to generate Equation (3):
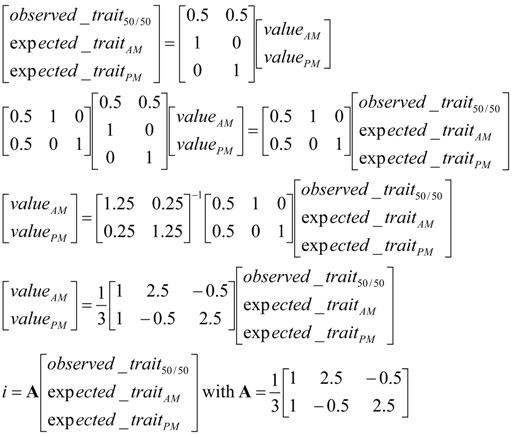
(3) where **i** is the vector that contains the AM and PM values that will be used to build the equations to predict daily yield from AM or PM data for the trait considered and A is the matrix containing the coefficients used to combine observed studied_trait_50/50_ with the expected_trait_AM or PM_, these values being equal to b × milk_yield_AM or PM_. The b coefficients for each studied traits were calculated based on regression analyses performed using Statistical Analysis System (SAS) software where the yield of the studied trait (calculated as content × milk yield) for the AM (PM) milking is the dependent variable and the milk yield observed after the AM (PM) milking is the independent variable.

The b coefficients were obtained from a second dataset that included 225,890 milk samples collected between October 2007 and February 2013 during the Luxembourg routine alternative milk recording (type T) from 31,510 cows (Holstein) in 491 herds (data T). During this milk testing, only one milk sample was collected per cow at one milking (AM or PM). Therefore, FAT_AM_ (FAT_PM_), SFA_AM_ (SFA_PM_), MUFA_AM_ (MUFA_PM_), UFA_AM_ (UFA_PM_), SCFA_AM_ (SCFA_PM_), MCFA_AM_ (MCFA_PM_), LCFA_AM_ (LCFA_PM_), and C18:1 *cis-9*_AM_ (C181 *cis-9*_PM_) were available for dataset T. This dataset also contained the AM or PM milk yield.

Based on this approach, expected AM and PM records were obtained for the dataset S. The daily average quantities (g/day) for all of the studied traits were estimated as the sum of yields after both milkings (AM and PM). Therefore, the final calibration dataset contained 79,971 records related to 51 traits [milk yield (expected AM, expected PM, and expected daily); fat content (expected AM, expected PM, and expected daily); fat yield (expected AM, expected PM, and expected daily; g/day); levels of seven different FAs or FA groups (expected AM, expected PM, and expected daily; g/dL milk), and the corresponding FA yields for these seven FA types/groups (expected AM, expected PM, and expected daily; g/day)].

#### 2.1.3. Validation Data

The equations to predict daily yields were validated using two distinct external validation datasets that included data for representative milk samples collected during two successive milkings in Luxembourg (between February and April 2013) and in the Walloon Region of Belgium (from October 2007 to June 2012).

The first validation dataset included representative milk samples (50 mL) collected from two consecutive milkings from 687 dairy cows (Holstein) belonging to 43 herds between February 2013 and April 2013 by CONVIS s.c. (Ettelbruck, Luxembourg; LUX data). This dataset contained observed yields from consecutive AM and PM milkings. Daily yields were also calculated. These samples were analyzed by MIR spectrometry using a FOSS Milkoscan FT6000 (Foss, Hillerod, Denmark). FA content (g/dL of milk) was estimated by applying the MIR calibration equations described in Table 1.

The second validation dataset included milk samples composed of 50% morning milk and 50% evening milk. These samples were collected from 138,141 Holstein cows belonging to 1291 herds that participated in the Walloon milk recording system from October 2007 to June 2012.Samples were collected from all of the cows milked in the herds on a given test day, and these samples were analyzed using MIR spectrometry (MilkoScan FT6000; FOSS, 2005) according to the normal milk recording procedure [11]. The final Walloon validation dataset contained 1,079,318 records (WAL data). AM and PM values were estimated by the same methodology used to create the calibration set.

### 2.2. Development of Statistical Models for Estimating Daily Yields from AM or PM Milking

Models were developed to investigate whether daily yields can be estimated by replacing the MI effect [10] with different traits that are easily recorded and that are related to changes in milk composition. Several variation factors were tested in order to build a robust model that uses information easily collected by milk recording organizations, including: stage of lactation (DIM), parity, yield traits (g/milking) during AM and PM milking, and the month of recording [14]. Stage of lactation is known to be one of the most influential factors affecting milk composition [10,15,16], and a month of recording was included in order to consider the season effect, and, indirectly, the feeding effect which affects the FA composition of milk [17,18]. Considering that not all of these influential factors may have statistically significant effects on all of the traits examined, an appropriate subset of variables for each model was determined using the stepwise GLMSELECT procedure in the SAS/STAT software package [19]. The data used to develop the models came from the calibration set. The TEST dataset required by the GLMSELECT procedure was the LUX validation dataset collected in Luxembourg and including real observed AM/PM data. The VAL dataset, required by the GLMSELECT procedure, corresponded to the WAL validation dataset which was collected in the Walloon Region of Belgium.This procedure allowed a model to be selected from the framework of general linear models. All of the models that were developed were compared for all of the studied traits: milk yield, FAT, SFA, MUFA, UFA, SCFA, MCFA, LCFA, and C18:1 *cis*-9.

The accuracy of the AM-PM predictions was evaluated using two criteria. First, root mean squared error (RMSE) was calculated (Equation (4)), which represents the SD of the difference between observed and estimated daily yields. The model with the smallest RMSE and the highest coefficient of determination (or correlation) was considered to provide the best fit.

(4) RMSE=SSEn−p

where n is the number of observations in the statistical model, p is the number of parameters (including the intercept), and SSE is the error sum of squares (*i.e*., the sum of the squared differences between each observation and its predicted value) for the estimated model.

The second criterion was R², defined as the coefficient of determination. The square root of this value is the correlation (Ry,ŷ) which represents the relationship between the observed and predicted values. Statistical parameters were calculated using the GLMSELECT procedure in the SAS/STAT software package [19].

A validation was applied on the best fitted model using the two available validation sets. The estimated statistical parameters were RMSE, the standard deviation of prediction (σŷ) and Ry,ŷ.

## 3. Results and Discussion

### 3.1. Available Data

Table 2, Table 3 and Table 4 present descriptive statistics of the traits studied. Daily average values showed the same direction for the three datasets except for milk production. The origins of each dataset could explain these differences. For example, the calibration dataset (Table 2) and the first validation set (Table 3) were obtained from Luxembourg, with the latter including milk samples that were collected over a short period of time (between February 2013 and April 2013). In contrast, the second validation dataset (Table 4) was generated from cows recorded in the Walloon Region of Belgium from October 2007 to June 2012.

**Table 2:** Descriptive statistics of the calibration dataset (N = 79,971).

Variable	Collection of milk sample	*Mean*	*SD*	*Min*	*Max.*	*Mean*	*SD*	*Min*	*Max*
Milk (kg/day)	AM	12.79	4.27	1.20	37.30				
	PM	13.57	4.41	1.20	39.20				
	Daily	26.36	8.33	2.40	72.80				
		g/dL milk				g/day			
Fat	Expected AM	4.11	0.71	1.01	7.23	515.31	164.29	22.13	1629.96
	Expected PM	4.55	0.78	1.12	8.00	605.64	191.21	24.48	1822.35
	Expected Daily	4.34	0.75	1.07	7.67	1120.94	340.33	46.61	3345.67
SFA	Expected AM	2.76	0.56	0.51	6.95	344.33	111.28	14.49	1272.59
	Expected PM	2.95	0.60	0.54	7.44	392.11	127.91	15.51	1268.89
	Expected Daily	2.86	0.58	0.53	7.21	736.41	229.39	30.00	2482.20
MUFA	Expected AM	1.15	0.24	0.29	3.84	144.14	53.26	6.08	678.89
	Expected PM	1.36	0.29	0.35	4.56	181.38	64.96	7.22	963.86
	Expected Daily	1.26	0.27	0.32	4.22	325.55	114.28	13.31	1562.22
UFA	Expected AM	1.34	0.27	0.37	4.25	168.57	61.34	7.14	765.02
	Expected PM	1.59	0.32	0.44	5.02	210.87	73.76	8.45	1047.49
	Expected Daily	1.47	0.29	0.40	4.66	379.44	130.42	15.59	1700.99
SCFA	Expected AM	0.38	0.08	0.10	1.02	47.77	16.50	1.63	200.32
	Expected PM	0.40	0.08	0.10	1.08	53.83	18.69	1.73	188.64
	Expected Daily	0.39	0.08	0.10	1.06	101.57	33.93	3.36	388.96
MCFA	Expected AM	2.16	0.47	0.06	5.77	268.89	87.51	6.98	975.09
	Expected PM	2.29	0.50	0.06	6.11	302.83	99.40	9.45	937.77
	Expected Daily	2.22	0.49	0.06	5.96	571.72	179.28	16.43	1891.53
LCFA	Expected AM	1.58	0.35	0.31	5.47	198.89	75.77	6.65	1028.49
	Expected PM	1.87	0.42	0.37	6.48	249.47	92.22	7.88	1352.77
	Expected Daily	1.73	0.39	0.34	6.01	448.39	162.72	14.53	2195.18
C18:1 *cis-9*	Expected AM	0.75	0.19	0.07	2.97	94.13	38.48	4.51	501.97
	Expected PM	0.91	0.23	0.09	3.59	120.85	48.53	5.46	777.79
	Expected Daily	0.83	0.21	0.08	3.30	214.98	84.65	9.96	1251.72

Min: minimum; Max: maximum; AM = morning milking; PM = evening milking, Daily = daily content; SFA = saturated fatty acids; MUFA = monounsaturated fatty acids; UFA = unsaturated fatty acids; SCFA = short chain fatty acids; MCFA = medium chain fatty acids; LCFA = long chain fatty acids.

In the calibration set, the average milk production between October 2007 and April 2013 (Table 2) was 26.36 kg/day, with 4.34 g fat/dL milk having a saturated part equal to 65.9%. Based on the WAL dataset, the average production was 24.11 kg milk/day, with 4.25 g fat/dL milk composed of 68.2% SFAs (Table 4). These values for fat and SFA content were slightly higher than those observed for the calibration set (Table 2). Overall, the quantities and content of individual FAs present in the milk samples and fat were consistent with those previously reported for the Walloon data [20,21,22]. The milk and fat yields had similar descriptive statistics compared to the results mentioned by Liu *et al.* [10] from their calibration set.

**Table 3:** Descriptive statistics of the Luxembourg (LUX) validation dataset (N = 687).

Variable	Collection of milk sample	*Mean*	*SD*	*Min*	*Max*	*Mean*	*SD*	*Min*	*Max*
Milk(kg/day)	AM	12.83	4.46	2.40	30.10				
	PM	15.13	5.18	2.80	33.00				
	Daily	27.96	9.41	5.20	57.00				
		g/dL milk				g/day			
Fat	AM	4.27	0.80	1.05	7.51	537.47	187.33	115.64	1257.44
	PM	4.68	0.80	1.59	7.51	695.33	239.95	165.56	1550.10
	Daily	4.49	0.71	2.33	7.33	1232.80	404.26	321.01	2656.58
SFA	AM	2.91	0.59	0.74	5.03	364.69	126.28	80.44	801.65
	PM	3.14	0.59	1.13	5.90	465.74	159.42	96.15	1075.73
	Daily	3.03	0.53	1.50	5.22	830.43	268.59	181.14	1698.26
MUFA	AM	1.18	0.29	0.27	3.67	148.79	61.37	29.71	448.47
	PM	1.33	0.30	0.39	3.37	198.17	85.18	52.20	763.26
	Daily	1.26	0.27	0.62	3.02	346.97	139.58	111.48	1094.06
UFA	AM	1.40	0.32	0.33	4.08	176.17	70.56	36.82	506.42
	PM	1.56	0.34	0.49	3.75	233.58	97.09	61.22	853.54
	Daily	1.49	0.30	0.75	3.37	409.75	159.88	128.22	1229.88
SCFA	AM	0.40	0.08	0.10	0.71	51.00	18.47	7.36	112.88
	PM	0.44	0.08	0.17	0.79	65.16	23.18	8.80	145.92
	Daily	0.42	0.07	0.24	0.71	116.16	39.48	16.16	240.74
MCFA	AM	2.30	0.47	0.56	3.95	288.14	98.17	53.54	622.87
	PM	2.47	0.47	0.94	4.11	365.12	121.71	67.72	799.04
	Daily	2.39	0.42	1.15	3.78	653.33	206.66	121.27	1314.99
LCFA	AM	1.66	0.41	0.46	5.29	209.24	87.77	48.90	669.326
	PM	1.86	0.44	0.55	5.09	278.76	122.40	71.67	1123.44
	Daily	1.77	0.39	0.80	4.49	488.00	200.03	147.86	1582.02
C18:1 *cis-*9	AM	0.81	0.23	0.21	2.85	102.13	46.85	23.20	361.59
	PM	0.91	0.24	0.27	2.54	136.21	65.34	35.44	593.22
	Daily	0.86	0.22	0.43	2.27	238.35	107.11	75.06	852.25

Min: minimum; Max: maximum; AM = morning milking; PM = evening milking, Daily = daily content; SFA = saturated fatty acids; MUFA = monounsaturated fatty acids; UFA = unsaturated fatty acids; SCFA = short chain fatty acids; MCFA = medium chain fatty acids; LCFA = long chain fatty acids.

**Table 4:** Descriptive statistics of the Walloon (WAL) validation dataset (N = 1,079,318).

Variable	Collection of milk sample	*Mean*	*SD*	*Min*	*Max*	*Mean*	*SD*	*Min*	*Max*
Milk (kg/day)	AM	11.41	4.27	0.20	49.00				
	PM	12.70	4.63	0.40	49.00				
	Daily	24.11	8.64	3.00	75.40				
		g/dL milk				g/day			
Fat	Expected AM	4.00	0.70	0.09	6.61	448.07	166.73	7.21	2730.67
	Expected PM	4.47	0.79	0.11	7.40	558.72	204.05	12.86	3418.45
	Expected Daily	4.25	0.75	0.10	7.22	1006.79	360.00	30.89	4640.37
SFA	Expected AM	2.78	0.56	0.00	5.35	311.57	119.42	0.62	1590.42
	Expected PM	3.00	0.60	0.01	5.79	375.79	142.26	0.42	1854.17
	Expected Daily	2.90	0.58	0.00	5.56	687.55	254.50	1.05	2792.34
MUFA	Expected AM	1.06	0.24	0.05	3.79	117.76	48.00	1.80	1022.04
	Expected PM	1.27	0.29	0.06	4.56	157.73	62.68	3.98	1458.89
	Expected Daily	1.17	0.27	0.06	4.18	275.49	107.92	8.62	1943.96
UFA	Expected AM	1.20	0.26	0.03	3.72	134.10	54.00	2.10	1127.60
	Expected PM	1.44	0.32	0.04	4.47	179.10	70.40	2.80	1588.10
	Expected Daily	1.33	0.29	0.03	4.10	313.10	121.30	5.50	2117.50
SCFA	Expected AM	0.36	0.08	0.01	0.93	40.79	17.00	0.67	191.11
	Expected PM	0.38	0.08	0.01	0.99	48.52	19.90	0.57	221.20
	Expected Daily	0.37	0.08	0.01	0.96	89.31	36.03	1.25	383.54
MCFA	Expected AM	2.18	0.49	0.00	4.49	243.85	94.77	0.63	1095.67
	Expected PM	2.36	0.53	0.01	4.86	294.22	113.42	0.43	1257.14
	Expected Daily	2.27	0.51	0.00	4.71	538.08	202.61	1.06	2125.97
LCFA	Expected AM	1.46	0.35	0.04	4.41	163.50	68.96	1.50	1503.15
	Expected PM	1.73	0.41	0.04	5.23	215.80	88.64	0.71	2066.22
	Expected Daily	1.60	0.38	0.04	4.83	379.30	153.91	2.21	2762.95
C18:1 *cis*-9	Expected AM	0.75	0.20	0.01	2.67	83.99	36.72	0.31	820.82
	Expected PM	0.89	0.23	0.02	3.17	110.90	47.33	0.29	1185.75
	Expected Daily	0.83	0.21	0.01	2.97	194.89	82.20	0.60	1585.61

Min: minimum; Max: maximum; AM = morning milking; PM = evening milking, Daily = daily content; SFA = saturated fatty acids; MUFA = monounsaturated fatty acids; UFA = unsaturated fatty acids; SCFA = short chain fatty acids; MCFA = medium chain fatty acids; LCFA = long chain fatty acids.

### 3.2. Phenotypic Correlations

Table 5 shows the correlations identified between AM and PM collection times, and for daily contents and yields, for all of the studied traits. Correlations between AM and PM values varied according to trait and were lower than one, suggesting that AM and PM records represent two distinct types of traits, and, therefore, need to have individual equations developed for estimating daily yield and content. Correlation values between yield traits were higher than those observed between content traits. For both units of expression, the correlations were lower for the fatty traits than milk yield. Moreover, for both content and yield traits, the PM milking records showed higher or similar correlation values with daily traits compared with the AM milking records. The same observation was done also by Berry *et al.* [13] from fat content and yield. However, Liu *et al.* [10] observed globally similar correlations between AM and PM values with a very slight tendency to have higher correlations for AM values.

Correlations between milking and daily yield traits varied from 92.4% (SFA; AM-DY) to 97.9% (milk yield; PM-DY). The strong positive correlations between daily and AM or PM yields observed in Table 5 suggest that it may be possible to estimate daily FA yields from AM or PM FA yields.

The FAT content and FAT yield correlation values were similar than those observed by Liu *et al.* [10]. These authors found correlation values equal to 59.0%, 86.4%, and 85.8% for AM/PM, AM/daily content (DC) and PM/DC correlations related to the fat content, respectively. The correlation values for the fat yield observed by these authors for the AM/PM, AM/daily yield (DY) and PM/DY were 83.9%, 92.7%, and 92.2%, respectively. Similar results were also obtained for milk yield. Liu *et al.* [10] found 90.8%, 97.9%, and 97.5% for AM/PM, AM/DY, and PM/DY correlations, respectively. The correlation values obtained by Berry *et al.* [13] were often lower than the ones found in this study. For fat content (yield), these authors calculated AM-PM, AM-DC, and PM-DC correlations equal to 36% (54%), 80% (84%), and 84% (90%), respectively. As observed in this study, the correlations related to fat yield were higher compared to the one observed for fat content. The same observation was done also by Liu *et al.* [10]. For milk yield, Berry *et al.* [13] found 85%, 97%, 95% for AM-PM, AM-DY, and PM-DY correlation values, respectively. The milk correlations between AM and PM values and between AM and PM values were slightly lower than the ones observed in this study but can be both considered as strong positive correlations. The differences in term of correlation values between Berry *et al*. [13] and Liu *et al*. [10] or our study can be probably explained by differences of herd management (feeding system), milking interval and milk production.

**Table 5:** Correlation values among morning (AM), evening (PM), and daily records for each studied trait expressed in g/dL of milk and kg/day. The values were obtained from LUX data (*i.e*., real observations, N = 687).

	g/dL of milk			kg/day		
Studied Trait	AM-PM	AM-DC	PM-DC	AM-PM	AM-DY	PM-DY
Milk				90.4	97.2	97.9
Fat	55.9	86.6	89.3	78.7	93.0	95.8
SFA	58.1	87.5	89.8	76.4	92.4	95.3
MUFA	63.7	88.3	92.1	80.9	93.3	96.6
UFA	63.3	88.3	92.0	81.4	93.6	96.6
SCFA	58.0	87.0	90.2	79.4	93.4	95.9
MCFA	60.8	88.3	90.6	76.4	92.5	95.2
LCFA	63.4	88.2	92.1	80.6	93.2	96.6
C18:1 *cis*-9	65.9	89.0	92.7	81.8	93.6	96.8

AM = morning milking; PM = evening milking; DC = daily content; DY = daily yield; SFA = saturated fatty acids; MUFA = monounsaturated fatty acids; UFA = unsaturated fatty acids; SCFA = short-chain fatty acids; MCFA = medium-chain fatty acids; LCFA = long chain fatty acids.

As also shown by Liu *et al*. [10] and Berry *et al*. [13] for milk fat, all correlations considered in Table 5 were lower for fatty traits compared to milk yield. This suggests that the prediction of daily yield or content from AM-PM records will be less accurate for fatty traits than milk yield.

### 3.3. Models Selected Using PROC GLMSELECT

Table 6 describes the equations that were selected using the GLMSELECT procedure for all of the studied traits. In other words, the models provided the best fit of data are described in Table 6. These models showed the smallest RMSE and the highest correlation between observed and estimated values. Based on these results, it appears that there were similarities between the effects included in the equations that used AM records and the ones included in the equations that used PM records for each studied trait. This observation suggests that PM and AM values had a similar evolution pattern but the differences came only from a question of scale. Indeed, PM values were always higher than AM values (Table 2, Table 3 and Table 4).

The PROC GLMSELECT procedure selected always combined effects. There were not individual effects such as only DIM or only lactation number in the selected equations. Such complexity of equations was not mentioned in previous studies [9,10,13]. However, Berry *et al*. [13] mentioned heterogeneous means and variances for 24-h yield over different parities, season of calving and DIM. Therefore, they realized 54 subclasses taken into account the parity, DIM and the season of calving. For all of these subclasses, they estimated the coefficients of regression. The same methodology was previously used by Liu *et al*. [10]. Based on the composition of selected equations mentioned in Table 6, this study confirmed this heterogeneity because separate regression coefficients were estimated following DIM, parity and month of test.

**Table 6:** Models selected by PROC GLMSELECT procedure.

Studied trait	Milking moment	Selected models
Milk	AM	a + b × DIM + c × month of test + d × (milk_AM × DIM × parity × month of test)
	PM	a + b × DIM + c × month of test + d × (milk_PM × DIM × parity × month of test)
Fat	AM	a + b × (qFAT_AM × milk_AM × DIM) + c × (parity) + d × (milk_AM × parity) + e×(qFAT_AM × milk_AM × parity)
	PM	a + b × (milk_PM × DIM) + c × (qFAT_PM × milk_PM × DIM) + d × parity + e × (qFAT_PM × milk_PM × month of test)
SFA	AM	a + b × (qSFA_AM × milk_AM × DIM) + c × parity + d × (milk_AM × parity) + e × (milk_AM × month of test)
	PM	a + b × (milk_PM × DIM) + c × (qSFA_PM × milk_PM × DIM) + d × (parity) + e × month of test
MUFA	AM	a + b × (milk_AM×DIM) + c × (milk_AM × parity) + d × (qMUFA_AM × milk_AM × DIM × parity) + e × (qMUFA_AM × milk_AM × month of test)
	PM	a + b × (milk_PM × DIM) + c × parity + d × (qMUFA_PM × milk_PM × DIM × parity) + e × (milk_PM × month of test)
UFA	AM	a + b × (milk_AM × DIM) + c × (qUFA_AM × milk_AM × DIM) + d × parity + e × (milk_AM × parity)
	PM	a + b × (milk_PM × DIM) + c × (qUFA_PM × milk_PM × DIM × parity) + d × (milk_PM × month of test) + e × (qUFA_PM × DIM × parity × month of test)
SCFA	AM	a + b × (qSCFA_AM × milk_AM × DIM) + c × parity + d × (milk_AM × parity) + e × month of test
	PM	a + b × (qSCFA_PM × milk_PM × DIM) + c × (milk_PM × parity) + d × (milk_PM × month of test) + e × (qSCFA_PM × DIM × parity × month of test)
MCFA	AM	a + b × (qMCFA_AM × milk_AM × DIM) + c × parity + d × (qMCFA_AM × milk_AM × month of test) + e × (qMCFA_AM × DIM × parity × month of test)
	PM	a + b × (milk_PM × DIM) + c × (qMCFA_PM × milk_PM × DIM) + d × (milk_PM × parity) + e × month of test + f × (qMCFA_PM × DIM × parity × month of test)
LCFA	AM	a + b × (milk_AM × DIM × parity) + c × (qLCFA_AM × milk_AM × DIM × parity) + d × (milk_AM × month of test) + e × (qLCFA_AM × milk_AM × month of test) + f × (qLCFA_AM × DIM × parity × month of test)
	PM	a + b × (milk_PM × DIM) + c × (qLCFA_PM × milk_PM × DIM × parity) + d × (milk_PM × parity × month of test) + e × (qLCFA_PM × DIM × parity × month of test)
C18:1 cis-9	AM	a + b × (milk_AM × DIM) + c × (milk_AM × parity) + d × (qC18:1 cis9_AM × milk_AM × DIM × parity) + e × (qC18:1 cis9_AM × milk_AM × parity × month of test) + f × (qC18:1 cis9_AM × DIM × parity × month of test)
	PM	a + b × (milk_PM × DIM) + c × (qC18:1 cis9_PM × milk_PM × DIM × parity) + d × month of test + e × (qC18:1 cis9_PM × milk_PM × month of test) + f × (qC18:1 cis9_PM × DIM × parity × month of test)

### 3.4. Goodness of Fit

Table 7 shows the correlation values calculated between the observed and estimated daily yields (Ry,ŷ), RMSE, and SDs of the daily yield predictions (σŷ) for each studied trait estimated from the milk samples collected during the AM or PM milking using the calibration set and the two available validation sets. The tested models were the models selected by PROC GLMSELECT and described in Table 6.

In order to appreciate the good fitting of a model, Liu *et al*. [10] indicated that σŷ should be close to the SD of the observed daily yield but must not be greater. In the present study, all of the estimates had smaller σŷ values than the observed SD values (Table 2, Table 3 and Table 4).

Except for milk yield, the observed correlations suggested that the estimations of daily yield were better when PM milking data were used. Indeed, the calibration correlation values were found to range from 96.4% to 97.6%, and from 96.9% and 98.3%, when estimations were realized from AM or PM milkings, respectively (Table 7). Except for milk yield, this is not in agreement with the observations made by Liu *et al*. [10] and Berry *et al*. [13]. However, the differences between AM and PM Ry,ŷ values were lower than 0.8%.

**Table 7:** Calibration and validation statistics (correlation values between true and estimate daily yield (Ry,ŷ), root mean square errors (RMSE) and standard deviation for each studied predicted trait (σŷ)) for the best model selected by PROC GLMSELECT. Ry,ŷ were expressed in % and RMSE and σŷ were expressed in kg/day for milk and g/day for the remaining studied traits.

Milking moment	studied trait	σŷ			RMSE			Ry,ŷ		
		Calib	LUX	WAL	Calib	LUX	WAL	Calib	LUX	WAL
AM	MILK	8.08	8.81	8.48	2.03	2.67	2.25	97.0	96.8	96.5
	FAT	328.00	385.88	350.93	90.52	160.62	98.47	96.4	92.7	96.2
	SFA	221.70	255.19	248.40	58.93	113.88	66.51	96.6	92.1	96.6
	MUFA	110.95	133.37	105.22	27.42	51.30	28.22	97.1	93.2	96.5
	UFA	127.50	152.31	118.81	32.40	58.14	32.36	96.9	93.4	96.4
	SCFA	32.95	37.34	35.37	8.10	15.90	8.58	97.1	93.1	97.1
	MCFA	173.37	198.66	195.92	45.66	89.12	52.81	96.7	92.0	92.7
	LCFA	158.29	188.74	151.19	37.72	73.34	38.71	97.3	93.3	96.8
	C18:1	82.63	105.13	82.50	18.53	38.66	20.11	97.6	97.0	93.4
PM	MILK	8.00	9.36	8.22	2.26	2.57	2.41	96.5	97.5	97.0
	FAT	331.70	405.91	352.63	85.49	124.65	91.27	96.8	95.6	97.1
	SFA	222.38	272.71	247.44	56.30	90.49	61.22	96.9	95.0	97.4
	MUFA	111.80	144.42	106.43	23.82	38.28	24.33	97.8	96.4	97.8
	UFA	127.39	165.01	119.82	27.91	43.56	27.98	97.7	96.5	97.7
	SCFA	33.01	40.71	35.09	7.85	13.01	8.06	97.3	95.5	97.7
	MCFA	173.77	207.82	198.22	44.13	70.49	49.00	96.9	94.8	97.4
	LCFA	159.35	208.85	151.48	32.95	55.78	33.06	97.9	96.4	97.9
	C18:1	83.21	109.27	79.94	15.54	27.81	16.41	98.3	96.7	98.1

Calib = calibration set including expected Luxembourg records (N = 79,971); LUX = Validation set including real collected Luxembourg records (N = 687); WAL = Validation set including expected Walloon records (N = 1,079,318); AM = morning milking; PM = evening milking; SFA = saturated fatty acids; MUFA = monounsaturated fatty acids; UFA = unsaturated fatty acids; SFCA = short-chain fatty acids; MCFA = medium-chain fatty acids; LCFA = long chain fatty acids.

Regarding the estimations of daily milk yield, the calibration correlation values were slightly lower than those obtained by Liu *et al*. [10] (e.g., 97.0% *vs.* 97.7% and 96.5% *vs.* 97.4% for the AM and PM milking data, respectively) (Table 7). The σŷ and RMSE values were also slightly higher in our study (for the AM and PM milking data: 8.08 and 8.00 kg/day *vs.* 7.85 and 7.83 kg/day for the σŷ values, respectively; and 2.03 and 2.26 kg/day *vs.* 1.72 and 1.84 kg/day for the RMSE values, respectively) (Table 7).

For the estimates of daily fat yield, obtained values for Ry,ŷ, and RMSE corresponded with a better fit of the model compared with Liu *et al*. [10] (for the AM and PM milking data: 96.4% *vs.* 94.3% and 96.8% *vs.* 94.0% for Ry,ŷ, respectively; and 90.52 *vs.* 106.0 g/day and 85.49 *vs.* 109.0 g/day for RMSE, respectively). The σŷ values were slightly higher in the present study (328.0 *vs.* 301.6 g/day and 331.7 *vs.* 300.6 g/day, respectively) (Table 7).

Better AM/PM predictions were observed for milk yield compared to fat content and yield. It was also observed by Liu *et al*. [10] and Berry *et al*. [13]. These last authors suggested that factors were missing in their equations permitting to predict AM/PM values for fat traits. However, in this study, the differences in terms of Ry,ŷ between milk and fat were lower. This is explained by a better fitting of fat traits in the current study.

Observed AM/PM calibration Ry,ŷ values for fatty acid traits were all within the same range and were higher than 96% suggesting a good prediction.

### 3.5. Model Validation

As expected, validation Ry,ŷ values obtained from the two validation sets were lower than calibration Ry,ŷ values. Validation RMSE values were higher than the observed calibration RMSE values (Table 7). However, RMSE observed for the LUX validation set (*i.e*., real observed data) were bigger than the WAL validation set (*i.e*., expected daily records). One hypothesis is that these differences were due to the initial step used to predict AM/PM values for the calibration set. A potential confirmation of this hypothesis comes from the fact that small differences were observed between the RMSE or Ry,ŷ observed from the first and second validation data sets for the equations predicting daily milk yield whose AM and PM milk records were always observed. However, the predictability stayed good with Ry,ŷ never lower than 92.0%.

Small differences observed between calibration and WAL validation results (*i.e*., these results were predicted using the same methodology as the one used for the calibration set) suggest a good robustness of the developed equations which was the main interest of the proposed methodology to build the calibration dataset. Indeed, as the first validation set which was composed of real records, was not large enough to cover the entire lactation, many parities, herds or cows, the theory of selection index was used to predict AM–PM records from 50% AM/50% PM collected records. Better results could be obtained by using only real observations but a large sampling procedure (larger than the one conducted for the LUX data) should be conducted to present a sufficient variability for DIM, parity, month of test as well as studied traits. The advantage of the selection index theory applied in this study is to use data routinely available at large scale to build the predictive models and, therefore, to require a smaller dataset containing real observations to validate the obtained models.

### 3.6. Milking Interval

The models proposed in the present study demonstrated that it is possible to estimate milk, fat, and FA yields without the use of MI recorded on site. To explain this observation, different regressions including the effects and covariates related to changes in milk were tested in order to estimate MI values (Table 8). An R² value of 0.86% was observed between MI and milk daily yield. Additional covariates and fixed classification effects can be included in the regression model (such as milk and fat yields obtained during one milking record) if we assume that the milk composition is also influenced by the MI due to the dilution effect. To predict daily yields for milk, fat, and protein, Berry *et al*. [13] introduced milk yield and fat yields of one milking a day. By using this approach, the obtained R² increased to 17.6% (Table 8).When the stage of lactation was added, the R² obtained was 18.2% (Table 8), while inclusion of the parity effect resulted in an R² value of 18.4%. All of the effects proposed to describe variations in MI were significant. Therefore, nearly 20% of the MI variability observed can be explained by a combination of effects related to milk composition and production. Consequently, we can assume that the MI effect can be partially replaced by a combination of data that are generally available and are easily recorded by milk recording organizations. In addition, the accuracy of reported MI can be problematic because, with increasing herd sizes and milking times, the actual MI for a given cow can be very different from the reported herd MI. Indirect predictors as used in this study have the advantage that they will be always known very precisely on an individual level.

**Table 8:** Regression coefficients (in %) for the regressions explaining the milking interval (MI) in function of milk production, fat (g/milking or /day), dim, and parity (N = 79,971).

MI	R^2^
Milk daily yield	0.86
Milk daily yield + Milk (AM or PM) yield	17.22
Milk daily yield + Milk (AM or PM) yield + FAT (AM or PM) (g/dL of milk)	17.64
Milk daily yield + Milk (AM or PM) yield + FAT (AM or PM) (g/dL of milk) + DIM	18.23
Milk daily yield + Milk (AM or PM) yield + FAT (AM or PM) (g/dL of milk) + DIM + parity	18.40

MI: milking interval; AM = morning milking; PM = evening milking; DIM = Days in milk.

## 4. Conclusions

The main objective of this study was to propose a practical, simple, and robust method for accurately estimating daily FA yields from a single milking (*i.e*., AM or PM milking). The obtained results show the interest to use the theory of the selection index to construct the calibration set in order to have more robust equations thanks to a large calibration set. With validation Ry,ŷ higher than 92% obtained from observed records for all studied traits, the results are promising, although further studies are needed to confirm these results by using a larger database. Moreover, the results obtained also shows that it is possible to replace the MI parameter with a combination of more reliable parameters such as: milk production and fat content, stage of lactation classes, the test month, and calving month. The application of the models developed in this study has the potential to reduce the number of collected samples per test-day (*i.e*., only one AM or PM sample is necessary instead of the two samples needed for the 50/50 sample), thereby reducing the costs associated with official milk recording (*i.e*., only one visit of the milk recorder in the farm), while still maintaining a high accuracy of predicted daily yields.

## References

[1] Arnould V.M.-R., Reding R., Bormann J., Gengler N., Soyeurt H. (2013). Review: Milk composition as management tool of sustainability. Biotechnol. Agron. Soc. Environ..

[2] Chilliard Y., Martin C., Rouel J., Moreau M. (2009). Milk fatty acids in dairy cows fed whole crude linseed, extruded linseed, or linseed oil, and their relationship with methane output. J. Dairy Sci..

[3] Van Haelst Y.N.T., Beeckman A., van Knegsel A.T.M., Fievez V. (2008). Short communication: Elevated concentrations of oleic acid and long-chain fatty acids in milk fat of multiparous subclinical ketotic cows. J. Dairy Sci..

[4] Gross J., van Dorland H.A., Bruckmaier R.M., Schwarz F.J. (2011). Milk fatty acids profile related to energy balance in dairy cows. J. Dairy Res..

[5] Rutten M.J.M., Bovenhuis H., Hettinga K.A., van Vanlenberg H.J.F., van Arendonck J. (2009). Predicting bovine milk fat composition using infrared spectroscopy based on milk samples collected in winter and summer. J. Dairy Sci..

[6] Soyeurt H., Dardenne P., Dehareng F., Lognay G., Veselko D., Marlier M., Bertozzi C., Mayeres P., Gengler N. (2006). Estimating fatty acid content in cow milk using mid-infrared spectrometry. J. Dairy Sci..

[7] Soyeurt H., Dehareng F., Gengler N., McParland S., Wall E., Berry D.P., Coffey M., Dardenne P. (2011). Mid-infrared prediction of bovine milk fatty acids across multiple breeds, production systems, and countries. J. Dairy Sci..

[8] Lee A.J., Wardrop J. (1984). Predicting daily milk yield, fat percent, and protein percent from morning or afternoon tests. J. Dairy Sci..

[9] Delorenzo M.A., Wiggans G.R. (1986). Factors for estimating daily yield of milk, fat, and protein from a single milking for herds milked twice a day. J. Dairy Sci..

[10] Liu Z., Reents R., Reinhardt F., Kuwan K. (2000). Approaches to estimating daily yield from single milk testing schemes and use of a.m.-p.m. records in test-day model genetic evaluation in dairy cattle. J. Dairy Sci..

[11] ICAR International Committee for Animal Recording, International agreement of recording practices. http://www.icar.org/documents/Rules%20and%20regulations/Guidelines/Guidelines_2012.pdf.

[12] Ouweltjes W. (1998). The relationship between milk yield and milking interval in dairy cows. Livest. Prod. Sci..

[13] Berry D.P., Olori V.E., Cromie A.R., Veerkamp R.F., Rath M., Dillon P. (2005). Accuracy of predicting milk yield from alternative milk recording schemes. Anim. Sci..

[14] Lock A.L., Garnsworthy P.C. (2003). Seasonal variation in milk conjugated linoleic acid and delta9-desaturase activity in dairy cows. Livest. Prod. Sci..

[15] Kelsey J.A., Corl A.B., Collier R.J., Bauman D.E. (2003). The effect of breed, parity, and stage of lactation on conjugated linoleic acid (CLA) in milk from dairy cows. J. Dairy Sci..

[16] Stoop W.M., Bovenhuis H., Heck J.M.L., van Arendonk J. (2009). Effect oflactation stage and energy status on milk fat composition of Holstein-Friesian cows. J. Dairy Sci..

[17] Arnould V.M.-R., Soyeurt H. (2009). Genetic variability of milk fatty acids. J. Appl. Genet..

[18] Chilliard Y., Ferlay A., Mansbridge R.M., Doreau M. (2000). Ruminant milk fat plasticity: Nutritional control of saturated, polyunsaturated, trans and conjugated fatty acids. Ann. Zootech..

[19] SAS (1999). SAS/STAT User’s Guide.

[20] Bastin C., Gengler N., Soyeurt H. (2011). Phenotypic and genetic variability of production traits and milk fatty acid contents across days in milk for Walloon Holstein first-parity cows. J. Dairy Sci..

[21] Bastin C., Berry D.P., Soyeurt H., Gengler N. (2012). Genetic correlations of days open with production traits and contents in milk of major fatty acids predicted by mid-infrared spectrometry. J. Dairy Sci..

[22] Soyeurt H., Gillon A., Vanderick S., Mayeres P., Bertozzi C., Gengler N. (2007). Estimation of heritability and genetic correlations for the major fatty acids in bovine milk. J. Dairy Sci..

